# Once Burnt, Twice Burnt: The Vulnerability of Insensate Flap Reconstructions

**DOI:** 10.7759/cureus.6569

**Published:** 2020-01-05

**Authors:** Sirke Rinkoff, Roshan Vijayan, Nora Nugent, Baljit Dheansa

**Affiliations:** 1 Plastic Surgery, Royal Free Hospital, London, GBR; 2 Plastic Surgery, Chelsea and Westminster, London, GBR; 3 Plastic Surgery, Queen Victoria Hospital, London, GBR; 4 Plastic Surgery, Queen Victoria Hospital, East Grinstead, GBR

**Keywords:** burn, reconstruction, insensate, flap

## Abstract

The occurrence of a burn injury in the same region as a previous burn is unusual outside the context of deliberate self-harm. Accidental burn injuries sustained to insensate flap reconstructions have previously been well reported in autologous breast reconstruction. Reports of such injuries in distant flap reconstructions of the hand and forearm are however unusual.

This case report describes a 40-year-old man who required a pedicled groin flap to reconstruct a burn injury on the ulnar border of his hand. Three years later, he suffered a burn to the same area whilst using an oven.

This case highlights the importance of counselling patients with insensate reconstructions regarding increased care and vigilance against inadvertent injury.

## Introduction

Insensate surgical reconstructions are frequently performed by plastic surgeons both in elective and trauma settings. They include free and pedicled tissue transfer for breast reconstruction post-mastectomy, and defects to the limb and head and neck regions after cancer excision or trauma. Accidental burn injuries sustained to insensate flap reconstructions have previously been well reported in autologous breast reconstructions through sunbathing, showering, spillage of hot fluids, and application of hot water bottles and heated packs [1,2]. Reports of such injuries in distant flap reconstructions of the hand and forearm are unusual; however, one report described burn injury after the use of heated packs to a free parascapular flap to the volar forearm [3]. The rarity of this description perhaps reflects the low number of upper limb soft tissue reconstructions performed and the high visibility of the hand, rendering protective behaviours more likely.

## Case presentation

**Figure 1:**
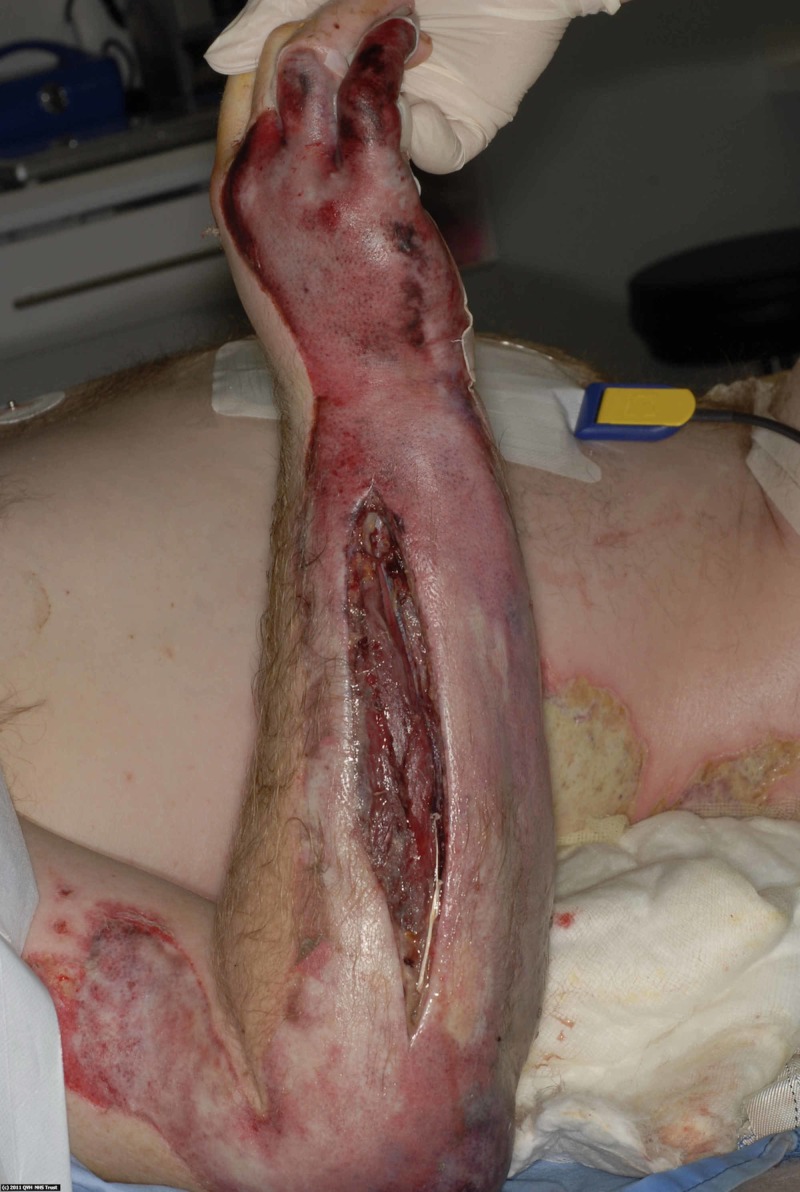
Initial burn injury, demonstrating the fasciotomy incision to the forearm for compartment syndrome.

This case describes a 40-year-old man with epilepsy who sustained 15% total body surface area scald burns to his back, buttocks and arms after suffering a seizure while in the bath. He was transferred to the regional burns unit where both forearms were found to have developed compartment syndrome and required fasciotomies (Figure 1).

**Figure 2:**
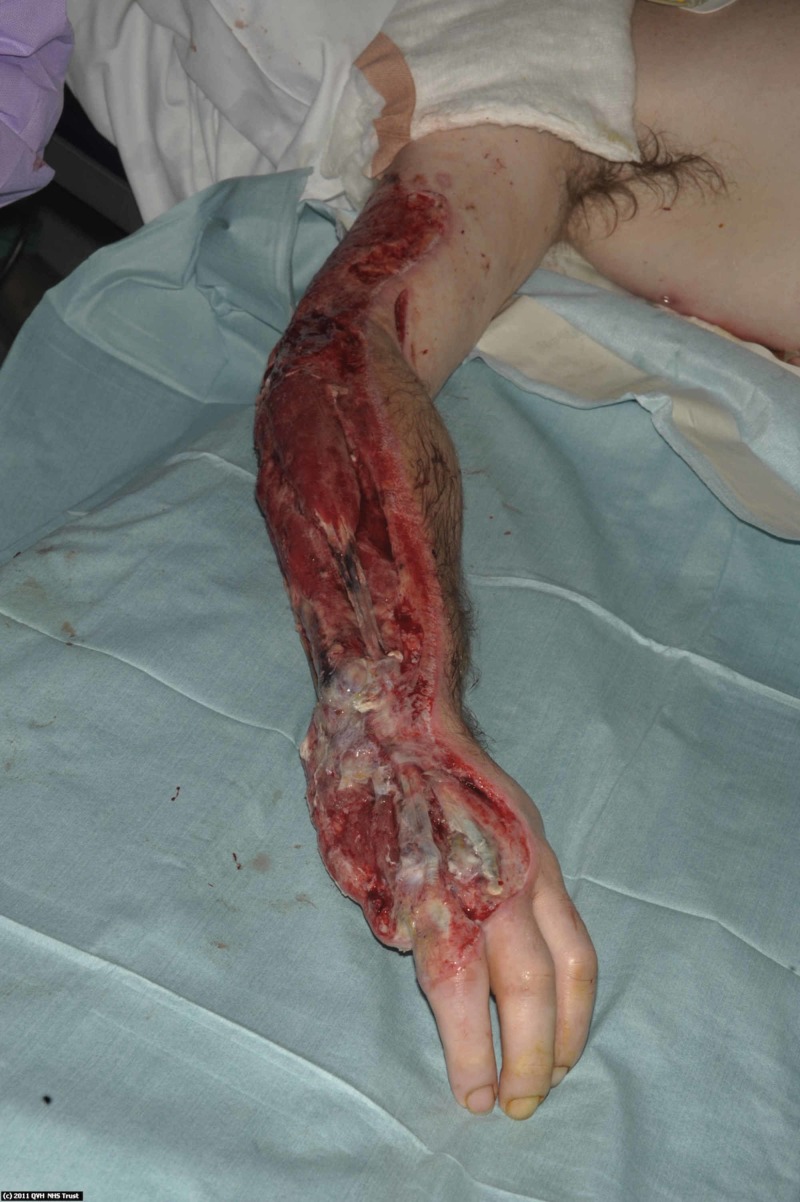
Post-debridement of the full thickness burns to the forearm, the elbow and wrist joints was exposed. The little finger required amputation.

**Figure 3:**
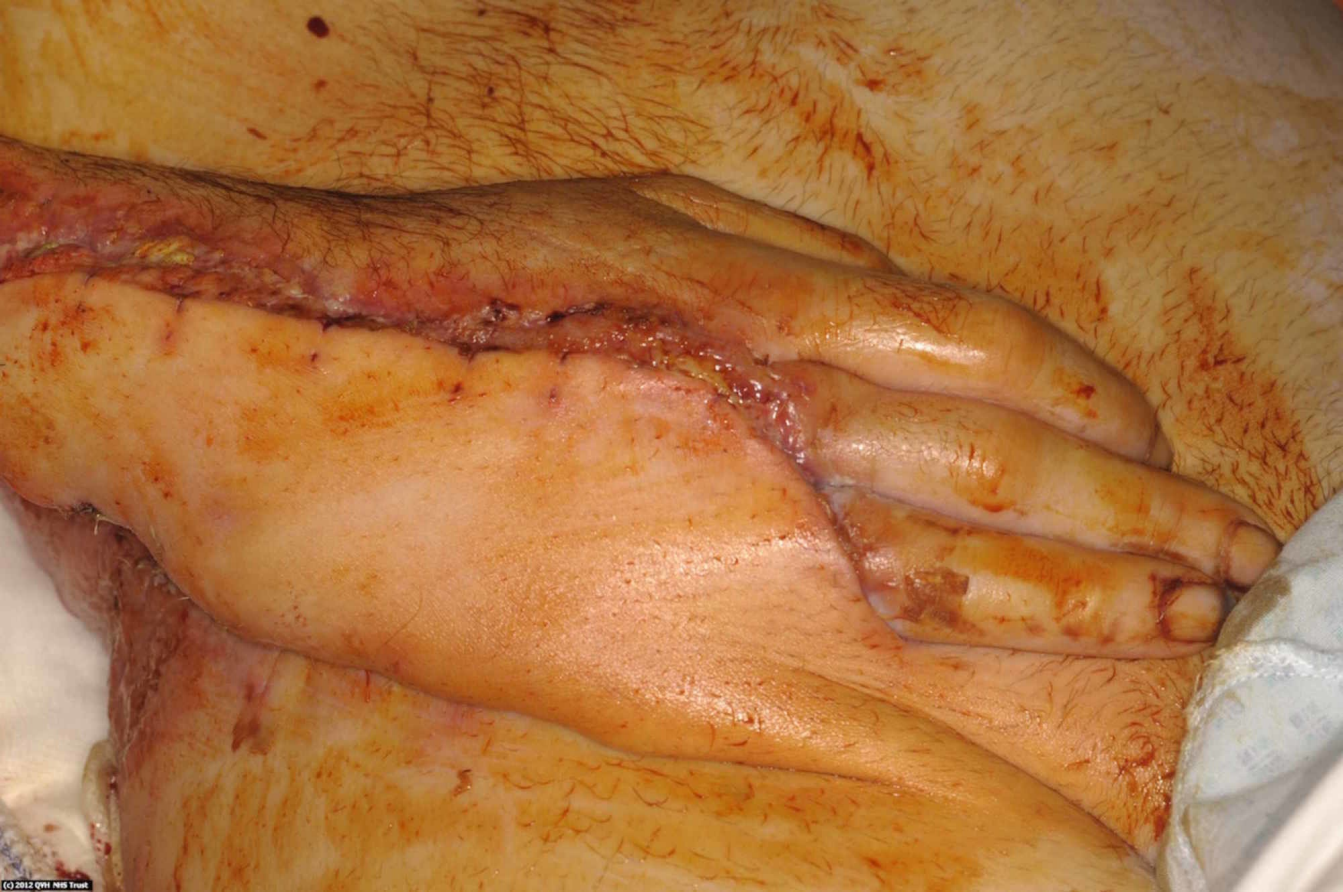
A pedicled groin flap was used for coverage of the hand wound. The pedicle was divided several weeks later.

Such was the extent of his injury that after surgical debridement he was left with significant soft tissue defects to his right upper limb (Figure 2) requiring complex soft tissue coverage. Exposure of his right elbow joint was managed with a medial arm flap. A large soft tissue defect over his ulnar wrist and hand required coverage with a pedicled groin flap (Figure 3).

Pedicled groin flaps are less commonly used since the development of microsurgical techniques for free tissue transfer; however, in this case the thermal injury made the vascular supply unreliable and therefore precluded free tissue transfer.

The interest in this case centres three years later, when the patient noticed an unusual appearance to the ulnar border of his hand overlying the previously formed flap, for which the patient attended his local accident and emergency.

**Figure 4:**
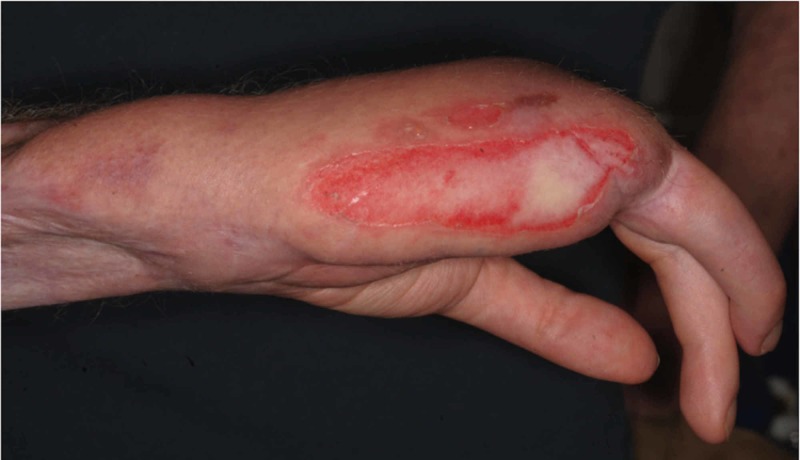
Deep partial thickness contact burn to the ulnar border of the patient’s reconstructed hand.

On examination, the patient was noted to have a small raw area on the ulnar border of his hand, which was otherwise asymptomatic (Figure 4). The patient was systemically well. The diagnosis was initially a mystery to the emergency department staff; however, the possibility of a burn was considered and a referral made to the regional Burns Unit. Assessment at the Burns Unit confirmed the diagnosis of a mostly deep partial thickness burn.

On reflection, the patient recalled that whilst removing potatoes from the oven a day prior, the ulnar border of his reconstructed right hand made contact with the hot glass door. He thought little of this initially, having no cutaneous sensation over the flap reconstruction, and thus experiencing no pain. 

**Figure 5:**
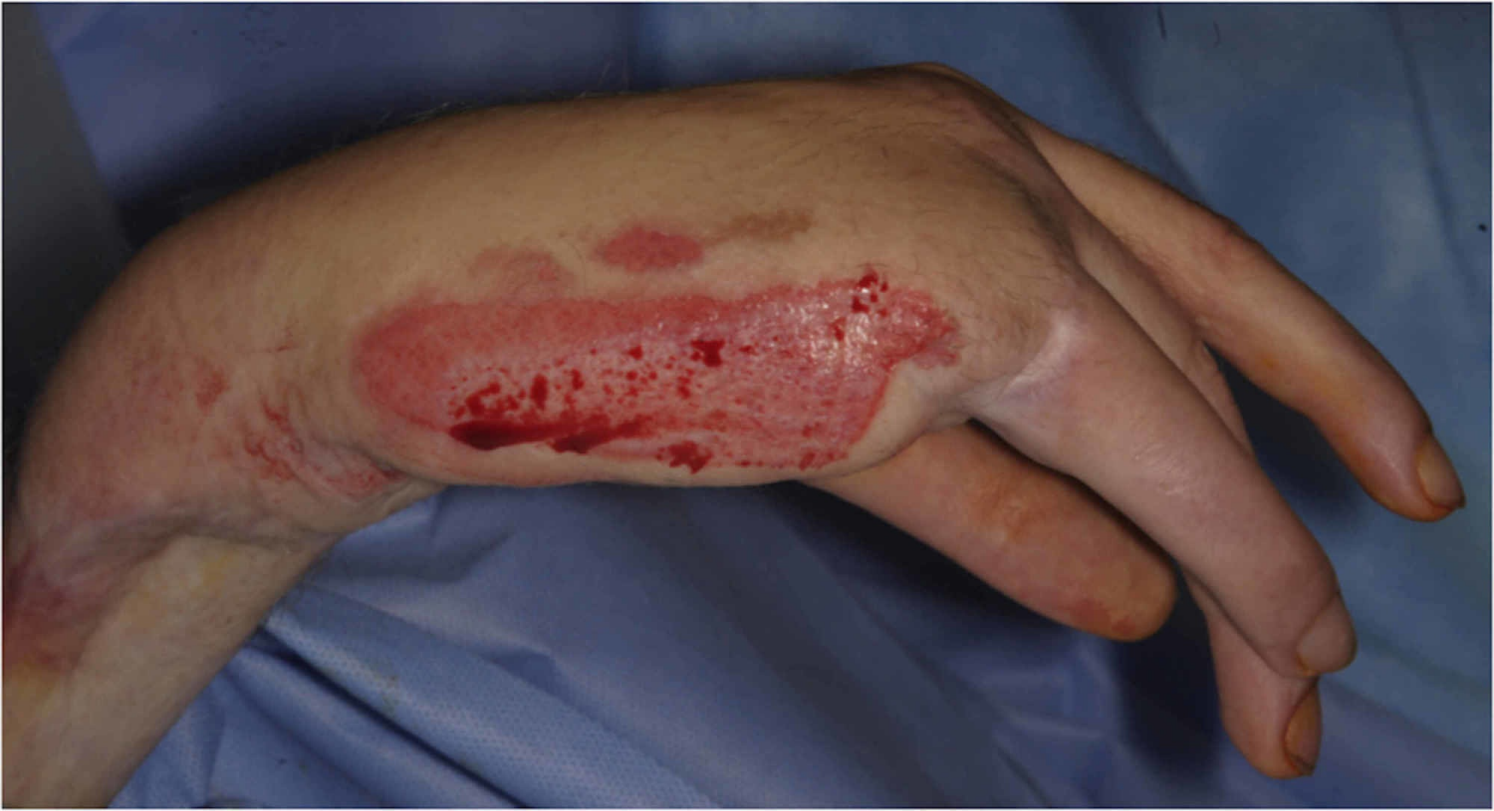
Post-debridement of the burn to viable dermis.

**Figure 6:**
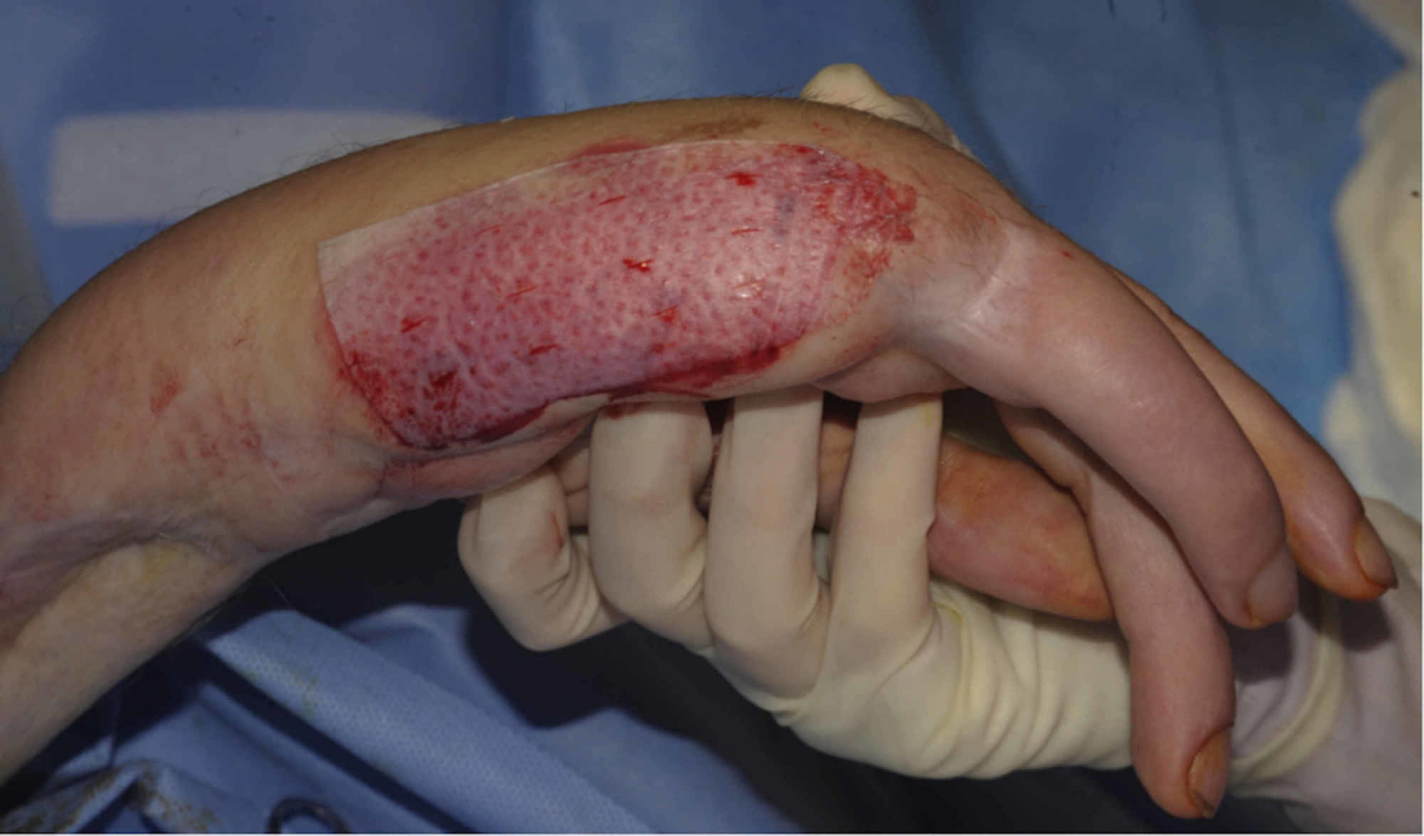
Application of a split thickness skin graft to the debrided wound.

**Figure 7:**
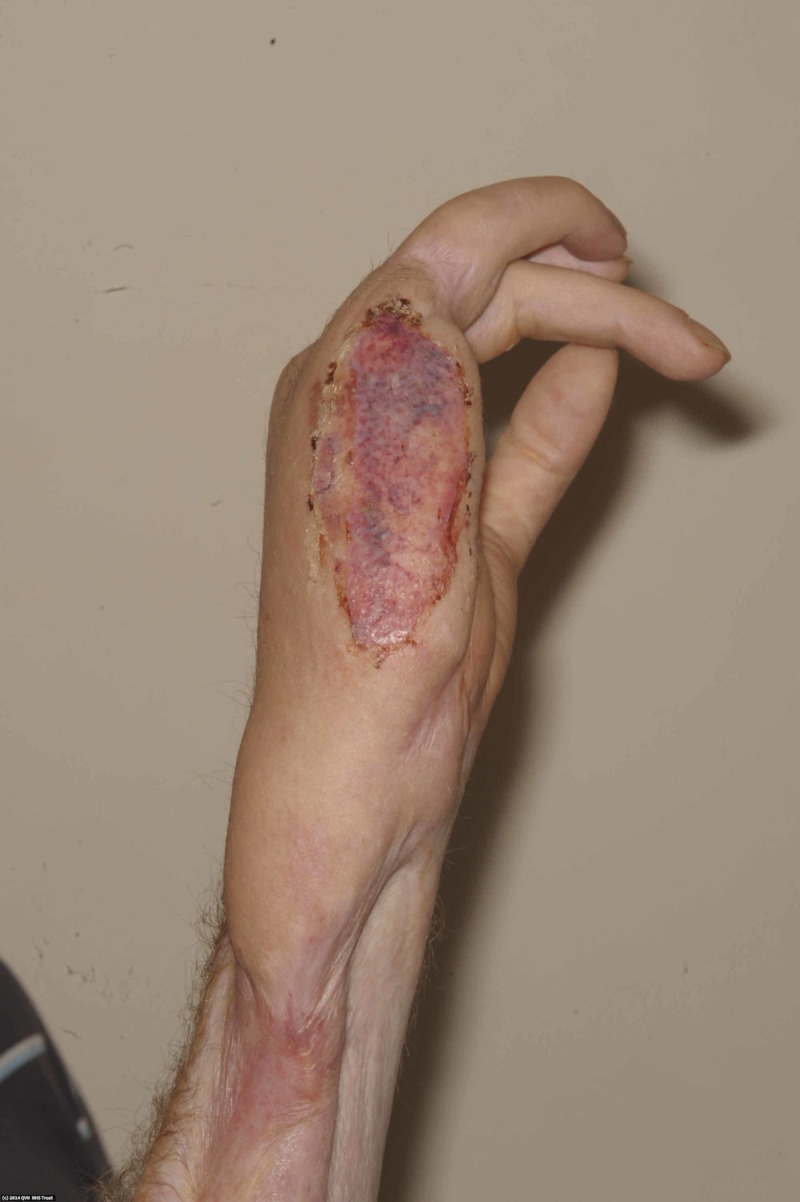
Ten days post skin grafting, the wound had almost fully healed.

Debridement of the burn by tangential excision was undertaken on the next scheduled operating list (Figure 5), and the defect covered with a split thickness skin graft (Figure 6), which healed well (Figure 7).

## Discussion

The occurrence of a burn injury in the same region as a previous burn is unusual outside of the context of deliberate self-harm. However, accidental burn injuries to insensate flaps have been described. The ulnar border of the hand is commonly exposed to surfaces during daily activities, and the fact that it is somewhat hidden and intuitively used to stabilize the hand against surfaces explains this flap’s relative vulnerability.

In this case, early surgical management was pursued as there is some evidence that healing in denervated tissue is impaired [4]. In order to avoid the complications of potentially protracted healing, the wound was promptly debrided and grafted. Whereas a sensate reconstruction with neurotized tissue would have been preferable, the extensive injury left no viable options for neurorrhaphy.

An unfamiliarity with flap reconstruction amongst clinicians not routinely exposed to this type of surgery, e.g., in primary care or the ED, can make the assessment and management more challenging. The unusual appearance of the patient’s hand made it difficult for those not familiar with flap reconstructions to differentiate what was normal from abnormal.

Just as with a diabetic patient who has peripheral neuropathy and presents with an asymptomatic infected ulcer on their foot, all clinicians should consider inadvertent injury, neglected wounds and infection in patients with insensate flaps. When assessing patients with unfamiliar surgical reconstructions, ask the patient to describe exactly what seems different to them. Where sensation over the reconstruction is ordinarily impaired for the patient, have a high index of suspicion for a neglected injury. Take a thorough history including asking about recent activities.

When encountering patients with reduced sensation over reconstructions, always remind them to be vigilant of the area, and to inspect regularly for injury, in a similar way that diabetics are advised regarding foot care. It is our duty to adequately inform our patients of the risks of insensate soft tissue, in order that we safeguard our patients and protect their surgical reconstructions.

## Conclusions

When assessing a patient with unfamiliar anatomy, a good history and examination is essential. This case highlights the importance of considering inadvertent injury in patients with insensate surgical flap reconstructions. We must also ensure that patients with insensate flaps are counselled about these risks also.
